# Changes in Alcohol Consumption among Different Population Groups during the SARS-CoV-2 Pandemic: Outcomes of the Slovenian Cross-Sectional National Survey (SI-PANDA)

**DOI:** 10.3390/ijerph192013576

**Published:** 2022-10-20

**Authors:** Sandra Radoš Krnel, Maja Roškar, Marjetka Hovnik Keršmanc, Maruša Rehberger, Gorazd Levičnik, Ada Hočevar Grom

**Affiliations:** 1 National Institute of Public Health, Analysis and Health Development Centre, SI-1000 Ljubljana, Slovenia; 2 National Institute of Public Health, Regional Unit Kranj, SI-4000 Kranj, Slovenia; 3 National Institute of Public Health, Health Data Centre, SI-1000 Ljubljana, Slovenia

**Keywords:** alcohol (ethanol), alcohol consumption (alcohol drinking), SARS-CoV-2 virus (SARS-CoV-2), pandemic (pandemics), vulnerable groups (vulnerable populations)

## Abstract

Background: Slovenia ranks amongst the countries with the highest recorded alcohol consumption. The mortality rate attributed to alcohol-related causes of death in Slovenia also exceeds the EU average. The aim of our research was to confirm the changes in alcohol consumption in Slovenia during the SARS-CoV-2 virus pandemic on a representative sample and to identify vulnerable groups at higher risk of increasing alcohol consumption. Methods: Two consecutive data collections of the National Survey on the Impact of the Pandemic on Life, each in different epidemiological situations, were conducted. A structured questionnaire was used to monitor the number of alcoholic beverages consumed during the pandemic, compared to the time before the pandemic. Results: The majority of the population did not change the number of alcoholic beverages consumed, and among those with changes, there were significantly more of those who drank less than those who drank more. Among respondents who drank a greater number of alcoholic beverages, statistically significantly higher proportions were found in younger age groups, people with post-secondary vocational education or higher, and people with a higher probability of mental health problems. Conclusions: During the pandemic crisis, we need to pay special attention to vulnerable groups that are at higher risk of increasing alcohol use.

## 1. Introduction

On a global scale, Slovenia ranks amongst the countries with the highest recorded alcohol consumption [1,2]. The registered consumption of pure alcohol per inhabitant aged 15 and over amounted to 9.8 L in 2020 [3]. In 2019, the EHIS survey (European Health Interview Survey), conducted by all European Union (EU) member states, ranked Slovenia above the average of EU countries in terms of the proportion of inhabitants, aged 15 and over, who reported having been drunk at least once a month [4]. The mortality rate attributed to selected alcohol-related causes of death in Slovenia also exceeded the EU average in the period of 2008–2017. In Slovenia, on average, three people die every day due to alcohol-related health problems [5,6].

In the first months of 2020, SARS-CoV-2 had been spreading all over the world. On 12 March 2020, an epidemic was declared in Slovenia. Numerous measures (including the closure of bars and restaurants) were adopted, and national lockdown was applied. These measures had a strong impact on personal and public life, with potential implications for the individual’s health [7,8,9,10].

Since the beginning of the SARS-CoV-2 virus pandemic, several studies have been conducted in which the authors studied possible changes in drinking habits during this period. In one of the larger studies from the first wave of the pandemic, in which 21 European countries participated, researchers measured the changes in alcohol consumption with a common variable, which contained information about the frequency and amount of drinking, as well as the frequency of binge drinking in the last month [9]. They found that in all countries (with the exception of two), alcohol consumption had declined, mainly due to a decrease in the frequency of binge drinking. Simultaneously, they detected a smaller reduction among respondents with lower personal income and those who reported experiencing stress due to the pandemic [9]. Additionally, data from the aforementioned study showed that approximately half of the respondents reported their drinking occasions not having changed in the past months, 26% of them reported a decrease in drinking, and 24% reported an increase. In addition to that, 23% of respondents reported a decrease in the amount of alcohol they had consumed on a single occasion, whilst 17% reported an increase. Respondents who reported a decrease in alcohol consumption were more likely to be male and of younger age. Interestingly, in Slovenia, during the first wave of the pandemic, researchers also found that respondents who reported having significant financial hardships had a fourfold higher risk of increased alcohol use, compared to those who reported milder hardships or none at all [9].

As noted by Panagiotidis et al. [11] and Rehm et al. [12], contemporary research suggested two possible mechanisms by which the SARS-CoV-2 virus pandemic impacted adult drinking behaviors. As a first mechanism at the beginning of the pandemic, they predicted an overall decline in alcohol consumption. This was subsequently confirmed in multiple studies due to the reduced physical availability and affordability of alcohol [9,11,12]. Affordability was reduced because of the decline in family incomes, whereas physical availability was a consequence of bar and restaurant closures, bans of alcoholic beverage sales in the evening hours, and also due to the ban of online alcohol sales. The decline in alcohol consumption was additionally being attributed to the fact that the SARS-CoV-2 virus pandemic is a health crisis, which can trigger self-protective behaviors in people and increase the likelihood of them following (at least to some degree) healthier lifestyle guidelines, which include a healthy diet, regular exercise, non-smoking, maintaining a healthy bodyweight, and limiting the use of alcohol [11]. Regarding the second mechanism, various authors [9,13,14,15,16,17] discussed the long-term effects of the pandemic, as they foresaw an increase in alcohol consumption, mainly due to the many negative impacts of the pandemic on mental health, since elevated levels of prolonged stress debilitate an individual’s ability to deal with everyday challenges and problems. This distress mechanism can be explained by considering alcohol use as a maladaptive coping strategy to manage psychological distress arising from an interplay of social isolation, insecurity, and financial difficulties [12,15,16,18]. In addition, previous literatures predicted an increase in alcohol consumption due to the reduced access to sources of help, including treatment services for alcohol use [11,12].

The issue of changes in alcohol consumption during the SARS-CoV-2 pandemic is even more complex; while a larger proportion of the population may report a decrease in alcohol use, it can mask an increase in the consumption reported by previously heavy-drinking individuals or other vulnerable groups [19,20,21].

In order to investigate and understand behavior, and to recognize, as well as address, the impacts of the pandemic among the residents of Slovenia, the National Survey on the Impact of the Pandemic on Life (SI-PANDA) was conducted. The aim of our research was to confirm the changes in alcohol consumption in Slovenia during the SARS-CoV-2 virus pandemic on a representative sample and to identify vulnerable groups at higher risk of increasing alcohol consumption.

## 2. Materials and Methods

### 2.1. Sample and Data Collection

The study on drinking behavior and possible changes in alcohol consumption among adult inhabitants of Slovenia during the SARS-CoV-2 virus pandemic was conducted within the framework of the National Survey on the Impact of the Pandemic on Life (SI-PANDA 2021) in two consecutive data collections [22]. The purpose of the survey was to investigate and understand the behavior, as well as to recognize and address the impacts of the pandemic among the inhabitants of Slovenia.

The SI-PANDA survey was conducted in two waves among 16,000 adult residents who lived in private households (not institutionalized). A probability sample was selected from this target population by the Statistical Office of Republic of Slovenia using sampling from the Central Population Register. The sample was stratified explicitly according to the size and type of settlement and implicitly according to the statistical regions. The total sample was divided into two subsamples to be used in two consecutive data collections. A group of 8000 selected residents were invited to participate in the survey at the time of epidemiologically more severe conditions and the other 8000 at the end of the spring, when less measures were in force. The first wave of data collection for the study on drinking behavior and possible changes in alcohol consumption was conducted between 25 January 2021 and 31 March 2021, and the second iteration of data collection occurred from 4 May 2021 to 18 July 2021.

Just before and still in January, when the first data collection started, very strict measures were in place in Slovenia. Schools were closed for pupils from the 6th grade onwards, as well as secondary schools and faculties. All public events and sporting activities were prohibited, along with a closure of restaurants, hotels, and shopping centers. Public transport was limited. Kindergartens were open only for emergency care. Movement restrictions between 9 p.m. and 6 a.m. were also enacted. With the start of the survey, some measures were gradually relaxed. At the beginning of February, all shops up to 400 m^2^ and primary schools for the first triad were opened. Gatherings of up to 10 people were permitted. At the beginning of March, all high school students returned to school, and student dormitories reopened. At the end of March, there was a change in the movement restriction to 22:00–5:00.

In May, when the second data collection started, all schools, including universities and higher education institutions, were opened. Hotels were able to operate again with 50% occupancy, and restaurant interiors were opened in all regions (with PCT condition), with limited operating hours between 5:00 a.m. and 10:00 p.m. In mid-May, it was allowed to hold cultural events with up to 50% occupancy of the seats, with the PCT condition and gathering of up to 50 people. High schools and colleges relaxed all measures. At organized public gatherings and events, there was no longer a gathering limit, only recommended physical distance. At the end of June, almost all services were opened again, and the time limit for the operation of bars and restaurants was lifted [23].

In both survey waves, in line with [24], a mixed-mode design was employed as a combination of ‘Computer-Assisted Web Interview’ (CAWI) and ‘Paper-and-Pen postal survey’ (PAPI). All selected persons received a notification letter and the unique password to access the online survey. The online survey was available to the selected participants for the entire duration of the study. Paper questionnaires were printed and sent with a postage-paid return envelope to all persons older than 54 and later to all non-respondents. A total of 6860 web and paper questionnaires were received from selected participants in both survey waves. Response rate was 48.9% in the first survey wave of data collection and 38.0% in the second. Data was weighted by gender, age group, and statistical region. The weighting was conducted for the entire population, with the reference date of 1 January 2021.

### 2.2. The Survey

The SI-PANDA survey addressed various topics that were regarded as significant during the SARS-CoV-2 virus pandemic. In this article, we focused on the question regarding the proportion of survey respondents who changed their habits in relation to alcohol consumption (“Have you changed your drinking habits in the last 12 months?”), shown in three response categories (drinking fewer alcoholic drinks or stopped drinking entirely; drinking more alcoholic drinks; and habits unchanged). The basic presentation of the impact of the pandemic on the consumption of alcoholic beverages was carried out with the help of socio-demographic and other explanatory variables, which were included in the survey or calculated from the basic variables: gender, age groups, attained level of education, activity status, marital status, household composition, financial situation in the last three months, infection with the new coronavirus, mental health, presence of at least one chronic disease, obesity, and being a current smoker [22].

### 2.3. Statistical Data Analysis

We utilized various statistical methods in order to determine the relationship between selected variables within an individual cross-sectional survey or to compare the results among the first and second data collections. To determine the correlation between sociodemographic and other explanatory variables with changes in alcohol consumption, the chi-square test (χ^2^) was established in each data collection, in line with previously used methodology [20]. In addition, the Bonferroni correction was used as a method to determine the statistical significance of differences between studied groups (sociodemographic and other explanatory groups).

Based on the test for the comparison of two comparable percentages, differences in the response percentages between groups within the same sociodemographic variable were analyzed (e.g., proportion of respondents without changes in drinking habits according to age groups—comparisons between all different pairs of age groups). Here as well, the Bonferroni correction was considered for the interpretation of differences and *p*-values. Statistical significance was set at *p* < 0.05. Lastly, we also compared the percentages of individual responses between the first and second cross-sectional survey within the same demographic groups with Bonferroni correction and the *p*-value set at *p* < 0.05. The article largely published data where the standard error of the share estimate was 5% or less, which meant that the estimate was sufficiently accurate and was published without restrictions. In case of a standard error of the estimate of more than 5%, the letter M was added to the proportion as a “less accurate estimate” [22].

## 3. Results

In both cross-sectional survey data collections, the majority (76.7% and 78.4%) of respondents reported not changing their alcohol consumption during the last 12 months; however, among the rest, there was a statistically significantly higher proportion of those who drank less, 19.1% (95% CI:17.6–20.6) and 16.7% (95% CI:15.1–18.4), compare to those who drank more, amounting to 4.3% (95% CI:3.6–5.1) and 4,9% (95% CI: 4.0–5.9) (Table 1). Amongst the respondents who reported having drank a greater number of alcoholic beverages, statistically significantly higher proportions were found in: (1) younger age groups, (2) respondents with post-secondary vocational education or higher, and (3) those with a higher probability of mental health problems. A statistically significantly lower proportion of retired respondents consumed more alcoholic beverages, compared to all other population groups, according to their activity status (Table 1).

The proportion of respondents who consumed fewer alcoholic beverages decreased statistically significantly between the first and second cross-sectional surveys (Table 2). These changes were observed in: (1) women, (2) respondents in the age group of 18 to 29 years, (3) respondents with secondary professional or technical education, (4) students, (5) those with married/extramarital relationships, (6) respondents living alone, (7) not living with children under the age of 18, (8) with the same financial situation as before the SARS-CoV-2 virus pandemic, (9) those who have been infected with the new coronavirus, and (10) respondents without mental health problems, without chronic diseases, without obesity, and are current smokers.

Conversely, the proportion of respondents who reported drinking more alcoholic beverages did not change statistically significantly between the first and second cross-sectional survey data collections. However, some population groups did consume more alcohol between the first and second data collections. A statistically significant increase in consumption was found among respondents with secondary professional or technical educations, those with the same financial situation as before the SARS-CoV-2 virus pandemic, among those who have not been infected with the new coronavirus, those with the likelihood of mental health problems present, and respondents who reported being current smokers.

## 4. Discussion

In between the two surveys data collections conducted, we found that the majority of respondents (77% in the first and 78% in the second iteration) did not change their alcohol consumption. Among those who reported changes in their alcohol consumption, there were significantly more of those who consumed lower amounts (19% in the first and 17% in the second data collection), compared to those who consumed higher amounts (4% in the first and 5% in the second iteration). In addition, the percentage of those who consumed fewer alcoholic beverages declined between the two survey data collections. This may be the result of the easing of certain measures, as described in more detail in the methodology section, which had been enacted to curb the spread of the SARS-CoV-2 virus in the period between the two cross-sectional survey data collections (e.g., the opening of restaurant outer gardens and terraces throughout Slovenia, which was followed by the opening of hotels and indoor restaurants, the lifting of restrictions on movement between 10:00 p.m. and 5:00 a.m. and also on regions, etc.). The release of the aforementioned measures consequently increased the possibility of socializing, as well as the availability of alcoholic beverages, which is, as noted by [25], an important factor for increased alcohol consumption. Decreasing and increasing the availability of alcoholic beverages during different waves of the pandemic can be seen as a “natural experiment” assessing the impact of reduced availability of alcohol-on-alcohol consumption. Therefore, these results should be considered by policymakers when deciding on implementing some specific actions of alcohol policy.

The data comparison concerning the percentage of the respondents who reported no changes, an increase, or a decrease in their alcohol consumption in the results of two other surveys conducted in Slovenia during the SARS-CoV-2 virus pandemic in 2020 showed certain differences; however, they also noted one similarity. In a survey from the first wave of the pandemic in 2020, in which 21 European countries participated, the percentage of respondents from Slovenia who did not change their habits related to alcohol consumption was around 60%, 23% of respondents reported a decrease in alcohol consumed on separate drinking occasions, and 17% reported an increase [10]. Moreover, in another study in which Slovenia had participated, the percentage of respondents who either reduced (36%) or increased (10%) their alcohol consumption was higher, compared to the results of our study [26]. The observed differences can be related to the chosen sampling methods, the time of the surveys, and also to the differences in the questions and the time intervals that the questions addressed. What all three studies conducted in Slovenia have in common is that the percentage of those who reported a decrease in their alcohol consumption during the SARS-CoV-2 pandemic was higher than the percentage of those who increased their alcohol consumption.

The results of various foreign studies, which observed changes in drinking habits during the SARS-CoV-2 virus pandemic, are not ubiquitous in this regard. Some, especially those conducted at the beginning of the pandemic, showed a greater decrease rather than an increase in alcohol consumption [15,20,21], the main reasons for this having been attributed to reduced availability of alcohol, which had occurred due to closed bars and restaurants, as well as physical distancing measures [11,12,17,27]. However, other studies, especially those conducted at later stages of the pandemic, have shown more frequent increases rather than decreases in the consumption of alcoholic beverages [13,14]. Increased levels of long-term stress could be a contributing factor to this, which in turn, may lead to people choosing ineffective coping strategies such as more frequently drinking and binge drinking, likely also due to the reduced availability of sources of help, including those aimed at treating problems with alcohol consumption [11,12].

The percentage of those respondents who consumed fewer alcoholic beverages declined between our two survey-data collections, but the difference was significant only for women. These results are consistent with another literature [28], stating that women are more vulnerable to stress related to the pandemic. Our findings are also consistent with the results of another Slovenian study [10] regarding drinking behaviors during the first wave of the SARS-CoV-2 virus pandemic, in which the authors found that women dominated among respondents who had increased their alcohol consumption in the last month before participating in the survey (amount and/or frequency of drinking alcohol). Even in general, other researchers noted that drinking alcoholic beverages as a coping mechanism for everyday problems and hardships is more characteristic of women rather than men [29]. This would also explain the general increase in risky or harmful alcohol consumption among women, which has also been commonly observed in Slovenia, especially among younger people [30].

Different population studies have also shown a higher prevalence of alcohol consumption among men, and those who are single, widowed or divorced, and those living by themselves [31,32,33,34]. In our research, however, it was shown that it was precisely these groups during the SARS-CoV-2 virus pandemic who reported to the highest extent that they drank fewer alcohol beverages. In another study, conducted in Slovenia during the first wave of the pandemic, it was also found that respondents who reported that their alcohol consumption had decreased during the pandemic were more likely to be male, [10] with similar findings also having been found in some other countries [35]. Foreign research has thus far found [28] that living in a household with children during the SARS-CoV-2 virus pandemic presents a risk factor for drinking, mainly due to increased stress when managing work from home, housework, and distance learning. Our results, however, are not entirely consistent with these findings. We determined that the percentage of those drinking fewer alcoholic beverages during the pandemic was lower in families with underage children, compared to families without underage children; the difference was significant only for the first iteration of our survey.

Regarding alcohol consumption and mental health, we found that during the pandemic, the percentage of those who consumed more alcohol than before the pandemic was higher among respondents with a high probability of mental health problems, compared to those without any mental health problems. This may suggest that among those with a high likelihood of mental health problems, the many negative mental health effects of the pandemic intensified, resulting in increased alcohol consumption as one of the inadequate coping mechanisms. Understanding the links between mental health and alcohol consumption during a pandemic is certainly an area which deserves further studying and monitoring, as it may help us develop appropriate policies and public health interventions to reduce alcohol-related harm [36].

The characteristics of respondents who reported an increase in the amount of consumed alcoholic beverages during the pandemic were of particular interest for our study, as they present a population group with increased risky behaviors in exceptional circumstances. We found that in both data collections of our survey, respondents younger than 50 years old, who had at least a post-secondary education, and respondents with a high probability of mental health problems stood out in this group. The opposite was observed among those in the population who drank more alcohol beverages, where there was a significantly lower percentage of retirees, compared to all other population groups, according to their activity status. The elderly and retired respondents could have had a greater degree of self-protective behavior, which may have led to these residents not increasing their alcohol consumption, as this group was at an increased risk of more severe consequences associated with the SARS-CoV-2 virus pandemic [37].

In the younger, most active population, which suffered the greatest lifestyle changes due to the SARS-CoV-2 virus containment measures, the reported increase in alcohol consumption may be the result of an inadequate way of coping with stress and the risks of losing a job, having to prolong their education, suffering financial losses, as well as increased stress when managing working from home, doing household chores, and supporting children who had to undergo distance learning [10,28,36]. Interestingly, respondents with the highest education, who otherwise had a lower percentage of abstainers and a higher percentage of those who got drunk at least once on a separate occasion in the last 12 months [38], according to Slovenian population surveys, increased their alcohol consumption during the pandemic. These findings are also similar to the study conducted by Rossow et al. [39], where the authors found that drinkers in the highest 10% for pre-pandemic consumption increased their drinking during the pandemic.

### Research Limitations

The study described in this article was conducted within the framework of the National Survey on the Impact of the Pandemic on Life, with the main purpose being to investigate and understand behavior, as well as to recognize and address the impact of the pandemic among the adult residents of Slovenia. The first data collection was performed in more severe epidemiological conditions, when stronger measures to prevent the SARS-CoV-2 virus spread were in force, in comparison to the second data collection, when fewer measures were in force. The interval between data collections was short, but regarding different epidemiological situations, still gave us insight towards behavior changes. Additionally, during time of the first data collection, governmental measures were rapidly changing; therefore, not all of the respondents answered the survey in the exact same conditions. Moreover, our data were obtained from the question “Have you changed your drinking habits in the last 12 months?” This period namely overlapped between the two data collections included in this research. The data were analyzed using stratified analysis; however, the covariance among certain variables could not be elucidated. Other limitations of the study included self-reporting of undesirable behaviors, which could cause issues of under-reported and subjective assessment of changes in consumption.

## 5. Conclusions

Our study showed that a higher share of the population consumed fewer alcoholic beverages, compared to the share of the population who consumed more alcohol during the pandemic, which confirmed the predictions of other authors regarding the decline in alcohol consumption, especially in the initial stages of the pandemic. As a possible mechanism of the impact of the SARS-CoV-2 virus pandemic on the drinking behavior of adults, many literatures highlighted the reduced physical availability and affordability of alcohol, as well as the fact that the SARS-CoV-2 virus pandemic is a health crisis which may trigger self-protective behaviors in people, thus increasing the likelihood of them living a healthier lifestyle. This should serve as an important message for decision makers, namely as reduced availability of alcohol is regarded as one of the (most) effective measures of alcohol policy in reducing alcohol-related harm, making it an important consideration for the future as well. At the same time, it is important to emphasize that between the two survey data collections, the proportion of respondents who consumed fewer alcoholic beverages during the pandemic decreased, which may indicate the long-term negative effects of the pandemic, in terms of increasing alcohol consumption. In extraordinary circumstances, such as the SARS-CoV-2 virus pandemic, we must pay close attention to groups with a lower ability to face everyday challenges and problems, due to which they may choose ineffective coping strategies, consume alcohol more frequently, and partake in binge drinking. According to the results of our study, the particularly vulnerable groups that should be given special attention are women (in whom we noted an increase in drinking habits), people with mental health problems, and lastly, young adults.

## Figures and Tables

**Table 1:** Respondents who changed their drinking habits in the past 12 months and those who did not, reported in the first and second cross-sectional survey data collections; this includes the total and is according to the explanatory variables.

		First Survey Data Collection, Total (n = 3574)						Second Survey Data Collection, Total (n = 2904)					
		Habits Unchanged		Drinking Fewer Alcoholic Drinks or Stopped Drinking Entirely		Drinking More Alcoholic Drinks		Habits Unchanged		Drinking Fewer Alcoholic Drinks or Stopped Drinking Entirely		Drinking More Alcoholic Drinks	
		%	Comparison between Groups	%	Comparison between Groups	%	Comparison between Groups	%	Comparison between Groups	%	Comparison between Groups	%	Comparison between Groups
	Total	76.7		19.1		4.3		78.4		16.7		4.9	
	Gender	χ^2^ = 49.4; *p <* 0.0001						χ^2^ = 43.5; *p <* 0.0001					
(A)	Male	72.0	B	23.3	B	4.7		73.8	B	20.9	B	5.3	
(B)	Female	81.5	A	14.7	A	3.8		83.2	A	12.3	A	4.5	
	Age Groups	χ^2^ = 105.6; *p <* 0.0001						χ^2^ = 68.1; *p <* 0.0001					
(A)	18 to 29 years old	66.3	BCDEF	28.1	BCDE	5.6	EF	71.9	CDE	19.2		8.9	DEF
(B)	30 to 39 years old	75.3	AD	18.3	A	6.4	EF	76.5		14.9		8.6	DEF
(C)	40 to 49 years old	78.7	A	14.2	AEF	7.1	DEF	82.3	A	12.8	F	4.9	EF
(D)	50 to 59 years old	82.6	AB	14.2	AEF	3.3	CF	79.8	A	16.9		3.3	AB
(E)	60 to 69 years old	78.1	A	20.2	ACD	1.7	ABC	81.4	A	16.7		1.9	ABC
(F)	70 years old or older	77.5	A	21.6	CD	0.9	ABCD	77.9		20.5	C	1.6	ABC
	Education	χ^2^ = 62.4; *p <* 0.0001						χ^2^ = 40.8; *p =* 0.0001					
(A)	Primary school or lower	80.3	BC	18.3		1.5	BD	78.4		19.7	D	1.9	CD
(B)	Secondary general or vocational technical	73.7	AD	20.2	D	6.0	AC	78.7		19.1	D	2.2	CD
(C)	Secondary professional or technical education	74.6	AD	22.3	D	3.1	BD	78.0		16.2		5.8	AB
(D)	Post-secondary vocational education or higher	79.0	BC	14.2	BC	6.8	AC	78.8		13.7	AB	7.5	AB
	Activity status	χ^2^ = 75.8; *p <* 0.0001						χ^2^ = 58.2; *p <* 0.0001					
(A)	Employed, self-employed	77.8	B	16.5	BC	5.7	CE	79.3	BDE	14.6	BC	6.1	CDE
(B)	Student	64.1	ACE	30.0	AC	5.9	CE	70.6	ACE	21.8	A	7.6	CE
(C)	Retired	77.9	B	20.9	AB	1.2	ABDE	79.5	BD	19.1	A	1.4	ABD
(D)	Unemployed	74.9		20.1		5.0	CE	70.8^M^	AC	19.3		9.9	ACE
(E)	Other	80.7	B	19.3		0.0	ABCD	85.6	AB	13.3		1.1	ABD
	Marital status	χ^2^ = 14.7; *p =* 0.0069						χ^2^ = 25.6; *p =* 0.0003					
(A)	Single, widowed, divorced	72.7	B	22.4	B	4.8		73.0	B	20.5	B	6.5	B
(B)	Married, extramarital relationship	78.4	A	17.6	A	4.1		81.0	A	14.9	A	4.1	A
	Household I	χ^2^ = 3.2; *p =* 0.3055						χ^2^ = 13.9; *p =* 0.0089					
(A)	Lives alone	73.4	B	21.7	B	4.9		71.7	B	22.9	B	5.5	
(B)	Lives with others	77.1	A	18.6	A	4.3		79.5	A	15.6	A	4.9	
	Household II	χ^2^ = 22.2; *p =* 0.0007						χ^2^ = 5.6; *p =* 0.1693					
(A)	Lives with children aged 18 or lower	78.9	B	15.2	B	5.9	B	80.2		14.1	B	5.7	
(B)	Does not live with children aged 18 or lower	75.7	A	20.6	A	3.7	A	77.8		17.5	A	4.7	
	Personal financial status in the last 3 months	χ^2^= 38.7; *p <* 0.0001						χ^2^ = 7.6; *p =* 0.2681					
(A)	Better than before	71.4	B	24.1	BC	4.5		75.9		18.3		5.7	
(B)	Remained the same	78.7	AC	18.2	A	3.1	C	79.8	C	15.6		4.6	
(C)	Worse than before	74.2	B	18.5	A	7.2	B	75.3	B	19.1		5.6	
	Infection with the novel coronavirus	χ^2^ = 10.0; *p =* 0.0434						χ^2^ = 1.6; *p =* 0.6043					
(A)	No	77.9	B	18.0	B	4.1	B	78.3		16.3		5.4	
(B)	Yes	72.5	A	21.4	A	6.1	A	79.9		15.9		4.3	
	Mental health	χ^2^ = 27.4; *p =* 0.0010						χ^2^ = 75.6; *p <* 0.0001					
(A)	High probability of problems	72.4	C	19.0		8.6	BC	71.1	C	17.0		11.9	BC
(B)	Likelihood of problems	75.3		20.3		4.3	A	76.0	C	17.1		6.9	AC
(C)	No problems	78.4	A	18.1		3.5	A	83.2	AB	14.5		2.3	AB
	Presence of chronic diseases	χ² = 0.4; *p =* 0.8601						χ² = 4.7; *p =* 0.2118					
(A)	No disease	77.1		18.6		4.2		80.1	B	15.2	B	4.7	
(B)	At least one discovered at any point	76.3		19.4		4.3		77.0	A	18.0	A	5.0	
	Obesity	χ² = 4.3; *p =* 0.2021						χ² = 1.0; *p =* 0.7294					
(A)	Yes	79.0	B	17.9		3.1	B	76.9		18.0		5.1	
(B)	No	76.0	A	19.5		4.5	A	78.8		16.3		4.9	
	Current smoker	χ² = 4.6; *p =* 0.2334						χ² = 34.8; *p <* 0.0001					
(A)	No	77.6	B	18.0	B	4.4		79.4	B	16.6		4.0	B
(B)	Yes	74.4	A	21.3	A	4.3		74.1	A	15.6		10.3	A

**Table 2:** Changes in drinking habits, related to respondents’ alcohol consumption in the past 12 months, between the first and second cross-sectional survey data collections. This includes the total and is according to the explanatory variables.

	Habits Unchanged			Drinking Fewer ALCOHOLIC Beverages or Stopped Drinking Entirely			Drinking More Alcoholic Beverages		
	First Survey Data Collection (%)	Second Iteration (%)	Comparison between Data Collections	First Survey Data Collection (%)	Second Iteration (%)	Comparison between Data Collections	First Survey Data Collection (%)	Second Iteration (%)	Comparison between Data Collections
Total	76.7	78.4	○	19.1	16.7	↓	4.3	4.9	○
Gender									
Male	72.0	73.8	○	23.3	20.9	○	4.7	5.3	○
Female	81.5	83.2	○	14.7	12.3	↓	3.8	4.5	○
Age Groups									
18 to 29 years old	66.3	71.9	○	28.1	19.2	↓	5.6	8.9	○
30 to 39 years old	75.3	76.5	○	18.3	14.9	○	6.4	8.6	○
40 to 49 years old	78.7	82.3	○	14.2	12.8	○	7.1	4.9	○
50 to 59 years old	82.6	79.8	○	14.2	16.9	○	3.3	3.3	○
60 to 69 years old	78.1	81.4	○	20.2	16.7	○	1.7	1.9	○
70 years old or older	77.5	77.9	○	21.6	20.5	○	0.9	1.6	○
Education									
Primary school or lower	80.3	78.4	○	18.3	19.7	○	1.5	1.9	○
Secondary general or vocational technical	73.7	78.7	↑	20.2	19.1	○	6.0	2.2	↓
Secondary professional or technical education	74.6	78.0	○	22.3	16.2	↓	3.1	5.8	↑
Post-secondary vocational education or higher	79.0	78.8	○	14.2	13.7	○	6.8	7.5	○
Activity status									
Employed, self-employed	77.8	79.3	○	16.5	14.6	○	5.7	6.1	○
Student	64.1	70.6	○	30.0	21.8	↓	5.9	7.6	○
Retired	77.9	79.5	○	20.9	19.1	○	1.2	1.4	○
Unemployed	74.9	70.8^M^	○	20.1	19.3	○	5.0	9.9	○
Other	80.7	85.6	○	19.3	13.3	○	0.0	1.1	○
Marital status									
Single, widowed, divorced	72.7	73.0	○	22.4	20.5	○	4.8	6.5	○
Married, extramarital relationship	78.4	81.0	↑	17.6	14.9	↓	4.1	4.1	○
Household I									
Lives alone	73.4	71.7	○	21.7	22.9	○	4.9	5.5	
Lives with others	77.1	79.5	↑	18.6	15.6	↓	4.3	4.9	○
Household II									
Lives with children aged 18 or lower	78.9	80.2	○	15.2	14.1	○	5.9	5.7	○
Does not live with children aged 18 or lower	75.7	77.8	○	20.6	17.5	↓	3.7	4.7	○
Personal financial status in the last 3 months									
Better than before	71.4	75.9	○	24.1	18.3	○	4.5	5.7	○
Remained the same	78.7	79.8	○	18.2	15.6	↓	3.1	4.6	↑
Worse than before	74.2	75.3	○	18.5	19.1	○	7.2	5.6	○
Infection with the novel coronavirus									
No	77.9	78.3	○	18.0	16.3	○	4.1	5.4	↑
Yes	72.5	79.9	↑	21.4	15.9	↓	6.1	4.3	○
Mental health									
High probability of problems	72.4	71.1	○	19.0	17.0	○	8.6	11.9	○
Likelihood of problems	75.3	76.0	○	20.3	17.1	○	4.3	6.9	↑
No problems	78.4	83.2	↑	18.1	14.5	↓	3.5	2.3	○
Presence of chronic diseases									
No disease	77.1	80.1	○	18.6	15.2	↓	4.2	4.7	○
At least one discovered at any point	76.3	77.0	○	19.4	18.0	○	4.3	5.0	○
Obesity									
Yes	79.0	76.9	○	17.9	18.0	○	3.1	5.1	○
No	76.0	78.8	↑	19.5	16.3	↓	4.5	4.9	○
Current smoker									
No	77.6	79.4	○	18.0	16.6	○	4.4	4.0	○
Yes	74.4	74.1	○	21.3	15.6	↓	4.3	10.3	↑

M: less accurate estimate; statistically significant differences: *p* < 0.05. Changes between data collections: ↑ statistically significant increase; ↓ statistically significant decrease; ○ no statistically significant changes.
